# Clinical and pharmacological factors influencing serum clozapine and norclozapine levels

**DOI:** 10.3389/fphar.2024.1356813

**Published:** 2024-03-27

**Authors:** Anna Mach, Anna Wnorowska, Marcin Siwek, Marcin Wojnar, Maria Radziwoń-Zaleska

**Affiliations:** ^1^ Department of Psychiatry, Medical University of Warsaw, Warsaw, Poland; ^2^ Department of Affective Disorders, Jagiellonian University Medical College, Cracow, Poland; ^3^ Department of Psychiatry, Addiction Center, University of Michigan, Ann Arbor, MI, United States

**Keywords:** clozapine, norclozapine, therapeutic levels, toxic levels, psychotropic drugs

## Abstract

**Background:**

Clozapine (CLO) is a very effective antipsychotic, whose use is associated with dose-dependent risk of complications. Due to high interindividual variability in CLO metabolism, there is a need to identify factors affecting the blood concentrations of CLO and its active metabolite, norclozapine (NCLO).

**Methods:**

A total of 446 blood samples (collected from 233 women and 213 men, aged from 18 to 77 years) were included in this study and analyzed for CLO and NCLO concentrations. The patients were treated at a psychiatric hospital in Warsaw in the years 2016–2021. Serum CLO and NCLO concentrations were determined with high-performance liquid chromatography coupled to UV.

**Results:**

The following factors were shown to increase serum CLO and NCLO levels: higher CLO dose (*p* < 0.001), female sex (*p* < 0.001), nonsmoker status (*p* < 0.001), the use of more than two additional psychotropic drugs (only in the case of CLO; *p* = 0.046), concomitant use of beta-blockers (for CLO *p* = 0.049; for NCLO *p* < 0.001), and older age (for CLO *p* < 0.001; for NCLO *p* = 0.011). Despite the use of CLO at daily doses within the recommended range (200–450 mg), the evaluated serum CLO and NCLO levels were within the therapeutic ranges in only 37% and 75% of cases, respectively, with 5.6% of cases exceeding the CLO toxicity threshold.

**Discussion:**

The use of CLO at recommended doses does not guarantee achieving therapeutic concentrations of CLO or NCLO. Women and nonsmokers were at the highest risk of having toxic CLO levels.

## 1 Introduction

Clozapine (CLO) is an atypical antipsychotic, which is especially effective in controlling positive symptoms in treatment-resistant schizophrenia, improving cognitive function, and treating negative symptoms of schizophrenia (Remington et al., 2016). CLO is recommended as the gold standard in the management of treatment-resistant schizophrenia (Hasan et al., 2012). Meta-analyses revealed CLO to be the most effective antipsychotic, particularly in reducing general, positive, and negative symptoms (Samara et al., 2016; Siskind et al., 2016; Huhn et al., 2019). Apart from its excellent clinical effectiveness, CLO reduces suicidal behaviors and the use of psychoactive substances in patients with schizophrenia (Khokhar et al., 2018). On the other hand, the use of CLO is associated with the risk of developing serious side effects that are considered as dose—independent which include: agranulocytosis, myocarditis, cardyomiophaty, pulmonary embolism. However many clozapine - induced side effects are believed to be dose dependent. In this group most often are mentioned: seizures (covulsions), hypersalvation, sedation, liver damage (Abidi and Bhaskara, 2003; De Berardis et al., 2018; Mauri et al., 2018; Haidary and Padhy, 2024). Despite over 3 decades of extensive research, the mechanisms responsible for the unique clinical effects of CLO remain unknown. Some authors suggest that the wide-spectrum effect of CLO, as well as the risk of some undesirable effects of the drug, can be attributed to its active metabolite, N-desmethylclozapine (norclozapine, NCLO) (Costa-Dookhan et al., 2020). NCLO is formed via CLO N-demethylation in the liver, a process that is regulated mostly by CYP3A4 and CYP1A2 and, to a lesser extent, by CYP2C9, CYP2C19, and CYP2D6. NCLO is the main circulating metabolite of CLO, and is characterized by a longer half-life than that of the mother compound. Plasma NCLO levels range from 10% to 100% of the corresponding CLO levels (Khokhar et al., 2018). Moreover, NCLO and CLO have unique pharmacological profiles, with the former exhibiting higher affinity for 5HT1C and 5HT2 receptors and a comparable level of affinity for D2 receptors, in comparison with the latter (Weiner et al., 2004). Moreover, the two compounds exert opposite effects on the cholinergic system. CLO is a receptor M1, M3, and M5 antagonist, whereas NCLO is a strong partial agonist of those receptors (Molins et al., 2017).

Notably, CLO metabolism is subject to great individual variability (Raedler et al., 2008; Piwowarska et al., 2016). The rates of CLO metabolism may vary between up to 50-fold between individual patients (Couchman et al., 2010). This is caused by the fact that CLO is part of many metabolic pathways and a substrate for many enzymes (including CYP1A2, CYP3A4, CYP2C19, CYP2C8, FMO3, CYP2E1, CYP2C9, CYP2D6, and UDP-glucuronosyltranferase), some of which (e.g., 2C19 and 2D6) is characterized by considerable genetic polymorphism (Akamine et al., 2017; Wishart et al., 2018). Apart from polymorphism of the genes involved in CLO and NCLO pharmacokinetics and pharmacodynamics, other factors, such as the dose, sex, age, smoking, ethnicity, and other medications, may affect the serum level variability (Haring et al., 1989; Bowskill et al., 2012; Suhas et al., 2020; Zeng et al., 2022). Although a correlation between selected factors and CLO levels has been identified in earlier studies, there have also been repeated appeals to verify these findings (Wagner et al., 2020; Flanagan et al., 2023b).

Therapeutic drug monitoring (TDM) is a tool that allows the dosage of a drug to be adjusted to the individual patient by quantifying the serum concentration of the drug. TDM relies on analytical methods such as high-performance liquid chromatography coupled to UV (HPLC-UV) or mass spectrometry (LC-MS/MS), which have high accuracy and precision. These instruments are usually available in the laboratories of university hospitals or large medical centers. Considering the limited availability of CLO Therapeutic Drug Monitoring (TDM) in routine clinical practice, there is a need to identify predictive factors that may help optimize treatment. Therefore, the aim of our study was to assess the factors affecting CLO and NCLO levels.

## 2 Materials

We analyzed serum CLO levels in 446 patients, 233 (52.2%) of whom were females and 213 (47.8%) of whom were males, aged from 18 to 77 years (mean age 47.4 ± 14 years). The patients were treated at the Nowowiejski Psychiatric Hospital in Warsaw in the years 2016–2021. TDM is standard procedure during clozapine treatment at the center where the study was conducted.

A vast majority of the tested blood samples (n = 428, 96.0%) were from patients diagnosed with schizophrenia (F20). The remaining samples were from patients diagnosed with schizoaffective disorders (F25) (n = 9, 2.0%), mental disorders due to brain damage and dysfunction (F06) (n = 6, 1.3%), or acute and transient psychotic disorders (F23) (n = 3, 0.7%).

Most of the evaluated patients (n = 241, 54.0%) were declared smokers, whereas 155 (34.8%) were nonsmokers, and the remaining 50 patients (11.2%) had an unknown smoking status.

## 3 Methods

This study had been approved by the local ethics committee at the Medical University of Warsaw (approval No. AKBE/83/2021).

The diagnoses were established based on the World Health Organization’s International statistical Classification of Diseases and related health problems, 10th revision (ICD-10) (WHO, 1993).

The evaluated concentrations of both CLO and NCLO were based on analyzing blood samples collected from patients referred for TDM as part of their hospitalization at the study center in Warsaw. We included only patients hospitalized in an inpatient ward, so we excluded active use of psychoactive substances. The blood tests were conducted after each patient had been on a stable dose of the medication for at least on week (stabilization phase). The patients whose blood samples were analyzed were receiving CLO at a daily dose appropriate for their clinical condition. The data regarding the daily dose of CLO, smoking status, and concomitant treatment with other medications at the time of the study were obtained from the TDM referral form completed by the referring psychiatrist. Later, hospital records of these patients were analyzed, and yielded demographic data.

The therapeutic ranges for CLO and NCLO used in this study were adopted based on Arbeitsgemeinschaft für Neuropsychopharmakologie und Pharmakopsychiatrie (AGNP) guidelines, and were 350–600 ng/mL for CLO and 100–600 ng/mL for NCLO (Hiemke et al., 2011; Patteet et al., 2014; Hiemke et al., 2018; de Leon et al., 2022). Serum CLO levels of ≥1,000 ng/mL were considered to increase the risk of toxicity and obligating the testing laboratory to immediately notify the prescribing physician (Hiemke et al., 2018).

Serum CLO and NCLO levels were tested 12 (±2) hours after the last dose of the drug (trough concentration). Venous blood samples were collected into BD Vacutainer^®^ tubes with coagulation activator. The serum obtained from centrifuged whole blood samples was used for the study analysis. All analyses were performed immediately after collection. The serum CLO and NCLO levels were determined with a validated high-performance liquid chromatography (HPLC) method. The Shimadzu chromatographic system used in the study comprised a SPD-M10Avp diode array detector, two LC-10ADvp pumps, DGU-14A degasser, an SCL-10ADvp controller, an SIL-10ADvp autosampler, and a CTO-10ASvp column oven. Once internal standard (clobazam) was added to all serum aliquots of 250 μL, liquid–liquid extraction with chloroform and hexane mixture was used. After blowing off the solvents, the dry residue was dissolved in 250 µL mobile phase. Chromatographic analyses were carried out using isocratic conditions. Separations of the drug tested was performed on a column hypersil GOLD Phenyl (5 µm × 150 mm x 4.6 mm) by Thermo Scientific. The mobile phase consisted of 0.01 M monopotassium orthophosphate and acetonitrile, mixed in a ratio of 62:38 at pH 4.3. Chromatography system parameters were as follows: mobile phase velocity 1 mL/min; column oven temperature 30°C; injection volume 30 μL; and the fixed wavelength detector operating at 254 nm. The range of the calibration curve for both analytes was 25–2000 ng/mL. The limit of detection was 13.9 ng/mL for CLO and 13.5 ng/mL for NCLO. The inter- and intra-batch variation were <5% for CLO and <7% for NCLO. The laboratory where the blood tests were conducted participates in LGC Standards’ Therapeutic Drug Monitoring Scheme.

The statistical analysis of quantitative variables was conducted with the use of descriptive statistics, such as means, standard deviations, medians, and ranges. The Shapiro–Wilk test was used to test whether the distribution of the analyzed quantitative variables deviated from a normal distribution. If the analyzed distribution was normal, we used the *t*-test to verify the hypothesis of equality of the means for two groups; if the distribution was not normal, we used the nonparametric tests to compare independent groups, the Wilcoxon Rank Sum test for two and the Kruskal–Wallis test for more groups. Spearman correlations were used to measure the association between pairs of variables.

Relationships between categorical variables were evaluated with the use of contingency tables and the chi-square test or Fisher’s exact test for small sample sizes.

The logistic regression generalized linear model (GLM) was employed in multivariate analysis. The optimal model was selected based on the Akaike information criterion (AIC) statistic. Type III tests were used to calculate the significance of each of the effects specified in the model.

*p*-values of less than 0.05 were considered statistically significant.

Statistical analysis calculations were conducted with the use of SAS/STAT v.15.2.

## 4 Results

Table 1 presents the mean daily CLO dose and serum CLO and NCLO levels.

**TABLE 1:** Daily CLO doses and serum CLO and NCLO levels.

	N	Mean	SD	Median	Min	Max
Serum CLO levels [ng/mL]	446	508.9	319.4	453.5	42.0	1,753.0
Serum NCLO levels [ng/mL]	446	220.0	154.2	176.5	31.0	1,161.0
Daily CLO dose [mg]	446	378.3	151.8	375.0	100.0	900.0

Determination of serum CLO and NCLO levels was conducted after the patient had been on a stable dose for at least 1 week. There was no significant effect of the duration of stable-dose treatment on serum CLO (*p* = 0.518) or NCLO (*p* = 0.813) levels.

Therapeutic CLO levels of 350–600 ng/mL were detected in 161 (36.1%) of the evaluated cases. In the case of therapeutic NCLO levels, 339 (76.0%) of the evaluated samples yielded therapeutic levels of 100–600 ng/mL. In 158 tests (35.4%) the therapeutic levels of both evaluated compounds were optimal (Table 2).

**TABLE 2:** Serum levels of CLO and NCLO.

Serum CLO levels [ng/mL]	Serum NCLO levels [ng/mL]			
	<100	100–600	>600	Total
<350	90 (20.2%)	70 (15.7%)	0 (0%)	160 (35.9%)
350–600	3 (0.7%)	158 (35.4%)	0 (0%)	161 (36.1%)
>600	1 (0.2%)	111 (24.9%)	13 (2.9%)	125 (28.0%)
Total	94 (21.1%)	339 (76.0%)	13 (2.9%)	446 (100%)

Daily doses of CLO showed a significant correlation with serum CLO (r = 0.31, *p* < 0.001) and NCLO (r = 0.37, *p* < 0.001) levels.

Out of the 446 analyzed blood samples, 284 (63.7%) came from patients receiving the recommended by European Medicines Agency (EMA) (EMEA, 2002) daily CLO doses of 200–450 mg, 46 (10.3%) from patients receiving doses less than 200 mg, and 116 (26.0%) from patients receiving over 450 mg CLO daily.

Table 3 presents the distribution of serum CLO and NCLO level ranges stratified by daily CLO doses.

**TABLE 3:** Ranges of serum CLO and NCLO levels stratified by daily CLO doses.

Daily CLO dose (mg)	Serum CLO levels [ng/mL]			Serum NCLO levels [ng/mL]			Total
	<350	350–600	>600	<100	100–600	>600	
<200	27	11	8	22	23	1	46
	58.7%	23.9%	17.4%	47.8%	50.0%	2.2%	
200–450	118	105	61	68	213	3	284
	41.6%	37.0%	21.5%	23.9%	75.0%	1.1%	
>450	15	45	56	4	103	9	116
	12.9%	38.8%	48.3%	3.5%	88.8%	7.8%	
Total	160	161	125	94	339	13	446

Out of the 284 blood samples from patients receiving the recommended daily CLO dose, only 105 (37.0%) revealed therapeutic levels of CLO, whereas 118 (46.6%) showed serum CLO levels to be below, and 61 (21.5%) above, the therapeutic range.

Out of the evaluated samples that came from patients receiving CLO at 200–450 mg/day, therapeutic levels of NCLO were observed in 213 cases (75.0%). The remaining tests showed NCLO levels to be either below (n = 68, 23.9%) or above (n = 3, 1.1%) the therapeutic range.

There was a significant correlation between patient age and serum CLO (r = 0.16, *p* < 0.001) and NCLO (r = 0.12, *p* = 0.012) levels.

Serum CLO and NCLO levels were also affected by patient sex and smoking status. Detailed data have been presented in Table 4. In female subgroup, the serum levels of both assessed compounds were higher than those in the male subgroup (*p* < 0.001). Conversely, in the subgroup of smokers the serum levels of both compounds were lower than those in nonsmokers (*p* < 0.001).

**TABLE 4:** Serum CLO and NCLO levels stratified by sex and smoking status.

	Serum CLO levels [ng/mL]							Serum NCLO levels [ng/mL]					
	N	Mean	SD	Median	Min	Max	*p*-value	Mean	SD	Median	Min	Max	*p*-value
Females	233	571.2	321.1	501.0	99.0	1,753.0	<0.001	251.6	173.8	217.0	31.0	1,161.0	<0.001
Males	213	440.7	304.0	374.0	42.0	1,712.0		185.5	120.8	154.0	33.0	632.0	
Non-smokers	155	629.0	356.4	560.0	71.0	1,753.0	<0.001	277.2	194.0	230.0	33.0	1,161.0	<0.001
Smokers	241	469.7	285.4	426.0	42.0	1,712.0		198.2	118.4	170.0	31.0	625.0	

In 184 cases (41.3%), the analyzed blood samples came from patients who were receiving CLO only; the remaining 262 cases (58.7%) were patients who were additionally on other drugs, including other psychotropic agents (n = 251, 56.3%), beta-blockers (n = 44, 9.9%), angiotensin-converting enzyme (ACE) inhibitors (n = 7, 1.6%), or antidiabetic drugs (n = 15, 3.4%).

Serum CLO levels were significantly higher in patients receiving additionally beta-blockers (median 521.0 ng/mL; range 65.0–1,753.0 ng/mL) in comparison with those in patients who were not receiving drugs from this class (median 441.5 ng/mL; range 42.0–1,712.0 ng/mL) (*p* = 0.038). No such differences were observed for either ACE inhibitors (*p* = 0.935) or antidiabetic drugs (*p* = 0.508).

The analyzed blood samples were subdivided into four groups in terms of the number of psychotropic agents the patients were receiving at the time. In our study there were 195 (43.7%) blood samples from patients who received no other psychotropic agent apart from CLO. Other patients received additionally one, two, and three or more other psychotropic agents (n = 153, 34.3%; n = 74, (16.6%); and n = 24, (5.4%), respectively).

The details on the serum CLO and NCLO levels stratified by the number of psychotropic drugs have been presented in Table 5.

**TABLE 5:** Serum CLO and NCLO levels stratified by the number of psychotropic drugs taken.

	Serum CLO levels [ng/mL]							Serum NCLO levels [ng/mL]					
	N	Mean	SD	Median	Min	Max	*p*-value	Mean	SD	Median	Min	Max	*p*-value
Clozapine with no other psychotropic drugs	195	478.4	305.7	422.0	63.0	1,712.0		209.4	125.4	176.0	31.0	658.0	
One additional psychotropic drug	153	493.6	300.0	428.0	65.0	1,605.0	0.546	213.8	158.1	169.0	33.0	900.0	0.478
Two additional psychotropic drugs	74	555.6	357.5	478.5	42.0	1,753.0	0.118	243.1	205.7	182.0	50.0	1,161.0	0.598
Three or more additional psychotropic drugs	24	709.8	356.7	589.0	77.0	1,485.0	0.002	275.3	148.0	227.0	33.0	618.0	0.025

**FIGURE 1:**
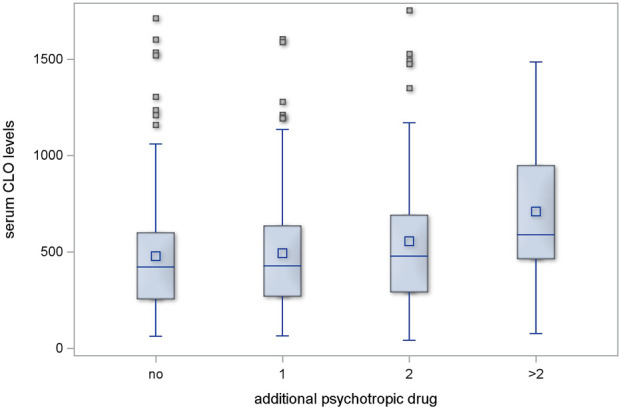
Serum CLO levels in subgroups receiving different numbers of concomitant psychotropic drugs.

Regimens with one additional psychotropic drug had no significant effect on serum CLO levels (*p* = 0.546); two additional psychotropic drugs had, likewise, no significant effect (*p* = 0.118). However, the serum CLO levels in the subgroup of patients who were receiving more than two additional psychotropic drugs showed a significant difference in comparison with those in those receiving no additional psychotropic drugs (*p* = 0.002). This is illustrated in Figure 1.

**FIGURE 2:**
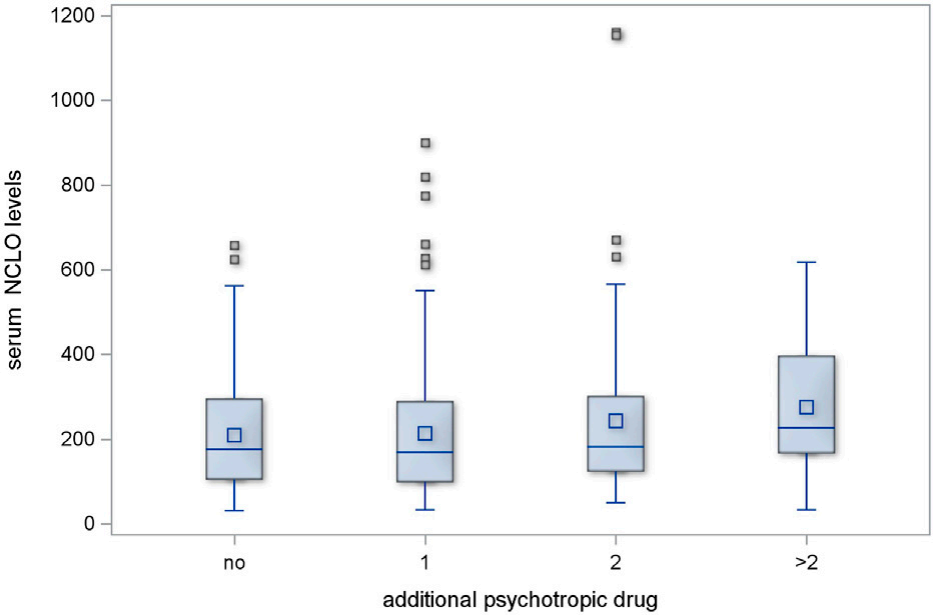
Serum NCLO levels in subgroups receiving different numbers of concomitant psychotropic drugs.

The regimens with one or two additional psychotropic agents produced no significant increase in serum NCLO levels (*p* = 0.478 and *p* = 0.598, respectively). However, the subgroup of patients who received more than two additional psychotropic drugs had significantly higher serum NCLO levels than patients receiving no concomitant psychotropic agents (*p* = 0.025). This is illustrated in Figure 2.

Out of the total 446 evaluated blood samples, 38 (8.5%) showed serum CLO levels equal to or greater than 1,000 ng/mL and 408 (91.5%) less than 1,000 ng/mL.

Toxic CLO levels were detected in 16 blood samples (5.6%) collected from patients receiving the recommended dose of the drug. In the subgroup of patients receiving CLO at doses above 450 mg/day, 22 persons (19.0%) had serum CLO levels indicating toxicity. Finally, there were no cases of toxicity-range serum CLO levels in the group receiving CLO at doses less than 200 mg/day (Table 6). There was a statistically significant relationship between the daily CLO range and drug levels indicating toxicity (*p* < 0.001).

**TABLE 6:** Toxic levels of CLO in subgroups receiving different daily clozapine doses.

Daily CLO dose (mg)	Serum CLO levels [ng/mL]		
	<1,000	≥1,000	Total
<200	46	0	46
	100.0%	0.0%	
200–450	268	16	284
	94.4%	5.6%	
>450	94	22	116
	81.0%	19.0%	
Total	408	38	446
	91.5%	8.5%	

There was no statistically significant difference between the ages of those patients whose blood samples showed toxic CLO levels and the ages of those whose serum CLO levels were below the toxicity threshold (*p* = 0.127).

The serum CLO levels of less than 1,000 ng/mL were detected in 206 blood samples (50.5%) collected from women and in 202 samples (49.5%) from men. Out of the blood samples with CLO levels of 1,000 ng/mL or higher, which indicates toxicity, 27 (71.1%) came from women and 11 (28.9%) came from men. These data show that toxic serum CLO levels were significantly more common in women (*p* = 0.015).

Out of the blood samples with serum CLO levels below the toxicity threshold, 132 (36.9%) came from nonsmokers and 226 (63.1%) came from smokers. Out of the blood samples whose CLO levels exceeded the toxicity threshold 23 (60.5%) were from nonsmokers and 15 (39.5%) came from smokers. Thus, CLO toxicity-range levels were significantly more common in nonsmokers (*p* = 0.008).

Statistical analysis showed no significant relationship between toxic serum CLO levels and the number of additional psychotropic agents (*p* = 0.316).

A multivariate analysis conducted to assess the factors affecting serum CLO levels included the following variables: the dose; sex; smoking status; one, two, or more additional psychotropic drugs; concomitant use of beta-blockers; and patient age (Table 7).

**TABLE 7:** Type III significance tests for the variables included in the model for CLO concentration.

Type III tests of effects				
Effect	Num DF	Den DF	F Value	*p*-value
CLO dose	1	387	59.92	<0.001
Sex	1	387	12.45	<0.001
Smoking	1	387	29.04	<0.001
Psychotropic drugs	3	387	2.69	0.046
Beta-blockers	1	387	3.87	0.049
Age	1	387	12.43	<0.001

Num DF, Den DF, the numerator and the denominator degree of freedom, F-Value—F, statistic.

The presented model demonstrated the following variables to affect serum CLO concentration:

• CLO dose—the linear dose-concentration relationship was statistically significant **(p < 0.001)**
• sex—the blood samples collected from women showed significantly higher serum CLO levels **(p < 0.001)**
• smoking—the blood samples collected from smokers showed significantly lower serum CLO levels **(p < 0.001)**
• the number of concomitant psychotropic drugs—the patients receiving more than two additional psychotropic agents had significantly higher serum CLO levels **(p = 0.046)**
• beta-blocker use—the blood samples from patients who were using these medications showed significantly higher serum CLO levels **(p = 0.049)**
• patient age—CLO concentrations showed a positive correlation with age, with the age–concentration relationship exhibiting statistical significance **(p < 0.001).**

A multivariate analysis conducted to assess the factors affecting serum NCLO levels included the following variables: CLO dose; sex; smoking status; the use of one, two, or more additional psychotropic drugs; concomitant use of beta-blockers; and patient age (Table 8).

**TABLE 8:** Type III significance tests for the variables included in the model for NORCLO concentration.

Type III tests of effects				
Effect	Num DF	Den DF	F Value	*p*-value
CLO dose	1	387	59.81	<0.001
Sex	1	387	16.47	<0.001
Smoking	1	387	32.12	<0.001
Psychotropic drugs	3	387	1.12	0.339
Beta-blockers	1	387	12.07	<0.001
Age	1	387	6.50	0.011

Num DF, Den DF, the numerator and the denominator degree of freedom, F-Value—F, statistic.

The presented model demonstrated the following variables to affect serum NCLO concentration:

• CLO dose—the linear dose-concentration relationship was statistically significant **(p < 0.001)**
• sex—the blood samples collected from women showed significantly higher serum NCLO levels **(p < 0.001)**
• smoking—the blood samples collected from smokers showed significantly lower serum NCLO levels **(p < 0.001)**
• the number of concomitant psychotropic drugs (the differences in concentrations between the subgroups were not statistically significant **(p = 0.339)**; this variable was included into the optimal model based on the Akaike information criterion
• beta-blocker use—the blood samples from patients who were using these medications showed significantly higher serum NCLO levels **(p < 0.001)**
• patient age—NCLO concentrations showed a positive correlation with age, with the age–concentration relationship exhibiting statistical significance **(p = 0.011).**

## 5 Discussion

In this study we evaluated the following factors affecting serum CLO and NCLO levels: the dose of CLO, sex, age, smoking status, the use of additional psychotropic drugs, and concomitant use of beta-blockers, ACE inhibitors, or antidiabetic drugs.

Daily CLO dosages showed a significant correlation with serum CLO and NCLO levels. The mean daily dose of CLO was 378.3 ± 151.8 mg, which falls within the therapeutic range (200–450 mg) recommended in the summary of product characteristics in Poland. This result was similar to those reported in other patients from Europe and Maori, but differed from the results obtained in Asian countries, where the mean daily dose of the drug is less than 300 mg/day (Menkes et al., 2018; de Leon et al., 2020). Caucasian patients required higher doses of the drug than Asian patients or patients from Mexico to ensure clinical effectiveness (Ng et al., 2005; González-Esquivel et al., 2011; Schoretsanitis et al., 2021). These differences support the effect of ethnicity on CLO pharmacokinetics.

CLO levels were within the therapeutic range in only 36.1% of cases, whereas NCLO levels fell within the therapeutic range in 76% of cases. Notably, only 35.4% of blood samples showed the concentrations of both compounds to be within the therapeutic ranges. The recommended concentrations were not reached in 20.2% of cases, which may contribute to a weaker clinical response. Conversely, in 2.9% of cases the recommended therapeutic ranges were exceeded, which increased the risk of toxicity. Similarly imperfect achievement of therapeutic concentration ranges has been also reported by other authors. Patteet et al. achieved the therapeutic range of CLO concentrations in only 22.3% of evaluated cases, with the remaining 67.9% of cases falling outside the desired range (Patteet et al., 2014). Such distribution of concentrations was also reported by British authors based on many-year-long monitored therapy; with 42.5% of samples showing drug concentrations below and 28.4% above the therapeutic range (Couchman et al., 2010).

In our study, we particularly focused on the samples collected from patients receiving CLO at a recommended daily dose. In this subgroup, only 37% and 75% of samples showed drug concentrations within the therapeutic ranges for CLO and NCLO, respectively, with 41.6% and 23.9% of samples failing to reach the therapeutic ranges for CLO and NCLO, respectively. Moreover, 21.5% and 1.1% of the analyzed blood samples exceeded the recommended therapeutic range for CLO and NCLO concentrations, respectively, with 5.6% of samples demonstrating CLO concentrations above the threshold for toxicity. These data unequivocally demonstrate the need to monitor blood CLO concentrations in each patient, to determine an effective and safe dose (de Leon et al., 2022).

In our study, patient age proved to be an important factor modulating serum CLO and NCLO levels, showing that the older the age, the higher the drug concentration. Serum levels of both compounds increased along with the age of patients who provided the analyzed blood samples. This phenomenon of drug concentrations increasing in older patients may be due to the age-related decreases in both the levels of CYP 450 isoenzymes (particularly CYP1A2 and CYP3A4 (Doki et al., 2009) and the clearance rates of CLO and NCLO (Ismail et al., 2012). Castberg et al. observed an increase in serum CLO levels by 108% at 80 years and by 197% at 90 years, in comparison with those in 40-year-olds (Castberg et al., 2017). These findings emphasize the clinical significance of CLO treatment monitoring in the eldest patients. This age group should be treated with increased caution due to the higher risk of achieving toxic drug concentrations.

Moreover, statistical analysis showed female patients to have higher concentrations of both compounds. This finding is consistent with those reported by other authors (Rostami-Hodjegan et al., 2004; Mayerova et al., 2018; Liu et al., 2023). The difference in drug concentrations between the sexes may be a result of lower CYT1A2 levels in women (Pollock, 1997; Scandlyn et al., 2008). Some authors suggested a possible inhibitory effect of both estrogens and oral contraceptives on CYP1A2 (Bigos et al., 2009). Other contributory factors may include a lesser blood perfusion through the liver, slower emptying of the stomach, or greater proportion of adipose tissue in women (Bigos et al., 2009; Smith, 2010; Mayerova et al., 2018; Liu et al., 2023). Due to CLO accumulation in adipose tissue, women have lower CLO clearance (Diaz et al., 2018). These data definitively demonstrate that sex is an important factor modulating blood concentrations of CLO and NCLO. The results of our study showed that the samples exceeding the toxicity threshold of CLO were statistically significantly more likely to have come from women than from men.

Another important factor affecting the evaluated drug levels was smoking. The samples collected from smokers yielded lower serum concentrations of both CLO and NCLO. Our findings are consistent with those observed in most of the previous studies on the effects of smoking on serum CLO levels (Wagner et al., 2020; Flanagan et al., 2023a), although there are also reports of the absence of such effects (Tang et al., 2007). The decreased serum CLO levels in smokers are associated with cytochrome CYP1A2 activation by multiring aromatic hydrocarbons present in tobacco smoke (Özdemir et al., 2001). CYP1A2 activation increases CLO clearance, which automatically reduces serum CLO and NCLO levels. In our study, the samples with toxic CLO levels were significantly more likely to have come from nonsmokers. Similar findings were reported by Flanagan et al., who observed the median CLO and NCLO concentrations to be by 36% higher in nonsmokers than in smokers (Flanagan et al., 2023a). Considering the fact that the proportion of smokers among people with schizophrenia is higher than that in the general population, smoking is one of the factors that may increase the risk of a relapse or inadequate repose to treatment (Rajkumar et al., 2013). On the other hand, smoking cessation or switching to electronic cigarettes may increase the risk of CLO toxicity (Cormac et al., 2010).

Evaluating the effect of additional psychotropic drugs on blood concentrations of CLO and NCLO, we compared the test results of those patients who received CLO alone with the test results of patients receiving one, two, or more additional psychotropic drugs. Receiving one or two additional psychotropic drugs had no significant effect on the serum concentrations of those two compounds. However, the patients receiving more than two additional psychotropic drugs had significantly higher CLO and NCLO levels than those of patients receiving CLO only. As mentioned above, CLO undergoes intensive hepatic metabolism with multiple CYP 450 enzymes. An addition of drugs that inhibit any of the enzymes, results in increased serum CLO and NCLO levels (Spina et al., 2016). Polypharmacy may produce a cumulative effect on various metabolic pathways and lead to multiple drugs competing for the same isoenzymes (Schoretsanitis et al., 2020). In such situations, TDM seems particularly reasonable as a potential tool in personalized treatment.

An estimated 10% of patients with schizophrenia also receive treatment with beta-blockers, such as metoprolol, propranolol, carvedilol, or nebivolol (Silva Gracia et al., 2017). Therefore, we assessed whether beta-blockers may affect serum CLO and NCLO levels. Both univariate and multivariate analyses showed that the use of beta-blockers increases blood concentrations of CLO and NCLO. This effect may be due to pharmacokinetic interactions. Both CLO and beta-blockers make use of the same CYP P450 isoenzymes in their metabolism (Perrild et al., 1989; Shin et al., 1999). One important example is the CYP2D6 isoenzyme, which becomes inactivated by both CLO and propranolol. Metoprolol may exert a similar effect; however, the data on its inhibitory effect on CYP2D6 are inconsistent (Polasek et al., 2011; Hefner et al., 2015). On the other hand, there have been reports of serious undesirable effects following combination treatment with CLO and metoprolol, which may be explained as a result of drug interactions at the level of CYP2D6 (Siwek et al., 2020). Moreover, since all evaluated medications are substrates for CYP2D6, the inhibitory effect of CLO, propranolol, or metoprolol may slow the metabolism of those drugs (Silva Gracia et al., 2017). This inhibitory effect should be less pronounced in the case of carvedilol, which is primarily metabolized by CYP1A2, and only to a lesser extent by CYP2D6 and CYP3A4 (Iwaki et al., 2016). Genetic polymorphism in CYP 450 genes may also play a role; however, they were not evaluated of this study.

Although CLO is mainly used in monotherapy, it is sometimes also used in combination with other drugs. In this year’s cross-sectional South-Eastern European study 70.5% of patients received CLO in polytherapy (Russo et al., 2023). The findings of our study suggest that, based on the pharmacokinetic and pharmacodynamic properties and the potential interactions of CLO, the use of one or two additional psychotropic drugs does not have to be associated with a high risk of toxicity. Moreover, we observed no significant relationship between toxic concentrations and the number of additional psychotropic drugs. Nonetheless the use of CLO in combination therapy may require particular caution and close TDM.

One of the strengths of our study is the fact that we evaluated the effects of concomitant psychotropic drug and beta-blocker use. This aspect was not evaluated in earlier studies, which was listed among those studies’ limitations (Liu et al., 2023). Available literature contains reports of known interactions with individual drugs or a group of drugs (Siwek, 2015). However, in medical treatment involving the use of CLO to manage treatment-resistant conditions, there is sometimes a need to use polypharmacotherapy. Another strength of our study is that it has increased the body of data on the active metabolite NCLO. Previous studies focused primarily on assessing the concentrations of the mother compound, CLO, itself and—occasionally—the CLO-to-NCLO ratio.

It may be advisable to conduct future studies based on more study groups to assess the additive effect of the factors mentioned above. Elderly female nonsmokers who receive combination medical treatment may be expected to be at a higher risk of CLO toxicity than young male smokers. Despite the fact that the analysis of our study data presented in this paper unequivocally indicates the greatest benefits in optimizing CLO treatment can be achieved via TDM, the factors listed in our paper may prove beneficial in everyday clinical practice.

It should be emphasized that TDM is an excellent tool of precision medicine for optimizing clozapine pharmacotherapy in terms of treatment efficacy and tolerability. Therefore AGNP guidelines highly recommend TDM in CLO treatment. The benefits of monitoring drug levels, especially in patients with insufficient treatment response to clozapine, far outweigh the costs associated with TDM.

## 6 Conclusion

1) The following evaluated parameters affected serum CLO levels: the dose; patient’s sex; smoking status; the use of one, two, or more than two additional psychotropic drugs; concomitant use of beta-blockers; and patient’s age.
2) The following factors affected serum NCLO levels: the dose of CLO; patient’s sex; smoking status; the use of one, two, or more than two additional psychotropic drugs; concomitant use of beta-blockers; and patient’s age.
3) The use of CLO at recommended doses does not guarantee achieving therapeutic concentrations of CLO or NCLO, and thus treatment effectiveness or safety.
4) In some cases, serum CLO levels exceeded the toxicity threshold despite the drug being used at recommended daily doses.
5) Serum CLO levels exceeded the toxicity threshold more commonly in women than in men and in nonsmokers than in smokers.

## Data Availability

The raw data supporting the conclusion of this article will be made available by the authors, without undue reservation.

## References

[B1] Abidi S. Bhaskara S. M. (2003). From chlorpromazine to clozapine—antipsychotic adverse effects and the clinician's dilemma. Can. J. Psychiatry 48, 749–755. 10.1177/070674370304801107 14733456

[B2] Akamine Y. Sugawara-Kikuchi Y. Uno T. Shimizu T. Miura M. (2017). Quantification of the steady-state plasma concentrations of clozapine and N-desmethylclozapine in Japanese patients with schizophrenia using a novel HPLC method and the effects of CYPs and ABC transporters polymorphisms. Ann. Clin. Biochem. 54, 677–685. 10.1177/0004563216686377 27932669

[B3] Bigos K. L. Pollock B. G. Stankevich B. A. Bies R. R. (2009). Sex differences in the pharmacokinetics and pharmacodynamics of antidepressants: an updated review. Gend. Med. 6, 522–543. 10.1016/j.genm.2009.12.004 20114004

[B4] Bowskill S. Couchman L. Maccabe J. H. Flanagan R. J. (2012). Plasma clozapine and norclozapine in relation to prescribed dose and other factors in patients aged 65 years and over: data from a therapeutic drug monitoring service, 1996-2010. Hum. Psychopharmacol. 27, 277–283. 10.1002/hup.2223 22419536

[B5] Castberg I. Westin A. A. Skogvoll E. Spigset O. (2017). Effects of age and gender on the serum levels of clozapine, olanzapine, risperidone, and quetiapine. Acta Psychiatr. Scand. 136, 455–464. 10.1111/acps.12794 28865402

[B6] Cormac I. Brown A. Creasey S. Ferriter M. Huckstep B. (2010). A retrospective evaluation of the impact of total smoking cessation on psychiatric inpatients taking clozapine. Acta Psychiatr. Scand. 121, 393–397. 10.1111/j.1600-0447.2009.01482.x 19824991

[B7] Costa-Dookhan K. A. Agarwal S. M. Chintoh A. Tran V. N. Stogios N. Ebdrup B. H. (2020). The clozapine to norclozapine ratio: a narrative review of the clinical utility to minimize metabolic risk and enhance clozapine efficacy. Expert Opin. Drug Saf. 19, 43–57. 10.1080/14740338.2020.1698545 31770500

[B8] Couchman L. Morgan P. E. Spencer E. P. Flanagan R. J. (2010). Plasma clozapine, norclozapine, and the clozapine:norclozapine ratio in relation to prescribed dose and other factors: data from a therapeutic drug monitoring service, 1993–2007. Ther. Drug Monit. 32, 438–447. 10.1097/FTD.0b013e3181dad1fb 20463634

[B9] De Berardis D. Rapini G. Olivieri L. Di Nicola D. Tomasetti C. Valchera A. (2018). Safety of antipsychotics for the treatment of schizophrenia: a focus on the adverse effects of clozapine. Ther. Adv. Drug Saf. 9, 237–256. 10.1177/2042098618756261 29796248 PMC5956953

[B10] De Leon J. Rajkumar A. P. Kaithi A. R. Schoretsanitis G. Kane J. M. Wang C. Y. (2020). Do asian patients require only half of the clozapine dose prescribed for caucasians? A critical overview. Indian J. Psychol. Med. 42, 4–10. 10.4103/IJPSYM.IJPSYM_379_19 31997860 PMC6970303

[B11] De Leon J. Schoretsanitis G. Smith R. L. Molden E. Solismaa A. Seppälä N. (2022). An international adult guideline for making clozapine titration safer by using six ancestry-based personalized dosing titrations, CRP, and clozapine levels. Pharmacopsychiatry 55, 73–86. 10.1055/a-1625-6388 34911124

[B12] Diaz F. J. Josiassen R. C. De Leon J. (2018). The effect of body weight changes on total plasma clozapine concentrations determined by applying a statistical model to the data from a double-blind trial. J. Clin. Psychopharmacol. 38, 442–446. 10.1097/JCP.0000000000000926 30106876 PMC6113094

[B13] Doki K. Homma M. Kuga K. Aonuma K. Kohda Y. (2009). Effects of CYP2D6 genotypes on age-related change of flecainide metabolism: involvement of CYP1A2-mediated metabolism. Br. J. Clin. Pharmacol. 68, 89–96. 10.1111/j.1365-2125.2009.03435.x 19660006 PMC2732943

[B14] EMEA (2002). SmPC leponex (clozapine) European agency for the evaluation of medicinal products. Available at: https://www.ema.europa.eu/en/documents/referral/summary-information-referral-opinion-following-arbitration-pursuant-article-30-council-directive/83/ec-leponex-associated-names-international-non-proprietary-name-inn-clozapine-background-inform_en.pdf .

[B15] Flanagan R. J. Hunter S. Obee S. J. (2023a). Effect of cigarette smoking on clozapine dose and on plasma clozapine and N-desmethylclozapine (norclozapine) concentrations in clinical practice. J. Clin. Psychopharmacol. 43, 514–519. 10.1097/JCP.0000000000001762 37930204

[B16] Flanagan R. J. Hunter S. Obee S. J. Reeves S. (2023b). Clozapine: dose, sex, ethnicity, smoking habit, age, body weight, and plasma clozapine and N -desmethylclozapine (norclozapine) concentrations in clinical practice. J. Clin. Psychopharmacol. 43, 131–138. 10.1097/JCP.0000000000001653 36735578

[B17] González-Esquivel D. F. Castro N. Ramírez-Bermúdez J. Custodio V. Rojas-Tomé S. Castro-Román R. (2011). Plasma levels of clozapine and norclozapine in Mexican schizophrenia patients. Arzneimittelforschung 61, 335–339. 10.1055/s-0031-1296207 21827043

[B18] Haidary H. A. Padhy R. K. (2024). “Clozapine,” in Disclosure: ranjit Padhy declares no relevant financial relationships with ineligible companies (Treasure Island (FL): StatPearls Publishing).

[B19] Haring C. Meise U. Humpel C. Saria A. Fleischhacker W. W. Hinterhuber H. (1989). Dose-related plasma levels of clozapine: influence of smoking behaviour, sex and age. Psychopharmacol. Berl. 99, S38–S40. 10.1007/BF00442557 2813665

[B20] Hasan A. Falkai P. Wobrock T. Lieberman J. Glenthoj B. Gattaz W. F. (2012). World federation of societies of biological psychiatry (WFSBP) guidelines for biological treatment of schizophrenia, part 1: update 2012 on the acute treatment of schizophrenia and the management of treatment resistance. World J. Biol. Psychiatry 13, 318–378. 10.3109/15622975.2012.696143 22834451

[B21] Hefner G. Unterecker S. Shams M. E. Wolf M. Falter T. Haen E. (2015). Melperone but not bisoprolol or metoprolol is a clinically relevant inhibitor of CYP2D6: evidence from a therapeutic drug monitoring survey. J. Neural Transm. (Vienna) 122, 1609–1617. 10.1007/s00702-015-1403-7 25940834

[B22] Hiemke C. Baumann P. Bergemann N. Conca A. Dietmaier O. Egberts K. (2011). AGNP consensus guidelines for therapeutic drug monitoring in psychiatry: update 2011. Pharmacopsychiatry 44, 195–235. 10.1055/s-0031-1286287 21969060

[B23] Hiemke C. Bergemann N. Clement H. W. Conca A. Deckert J. Domschke K. (2018). Consensus guidelines for therapeutic drug monitoring in neuropsychopharmacology: update 2017. Pharmacopsychiatry 51, e1–e62. 10.1055/s-0037-1600991 29390205

[B24] Huhn M. Nikolakopoulou A. Schneider-Thoma J. Krause M. Samara M. Peter N. (2019). Comparative efficacy and tolerability of 32 oral antipsychotics for the acute treatment of adults with multi-episode schizophrenia: a systematic review and network meta-analysis. Lancet 394, 939–951. 10.1016/S0140-6736(19)31135-3 31303314 PMC6891890

[B25] Ismail Z. Wessels A. M. Uchida H. Ng W. Mamo D. C. Rajji T. K. (2012). Age and sex impact clozapine plasma concentrations in inpatients and outpatients with schizophrenia. Am. J. Geriatr. Psychiatry 20, 53–60. 10.1097/JGP.0b013e3182118318 21422906

[B26] Iwaki M. Niwa T. Bandoh S. Itoh M. Hirose H. Kawase A. (2016). Application of substrate depletion assay to evaluation of CYP isoforms responsible for stereoselective metabolism of carvedilol. Drug Metabolism Pharmacokinet. 31, 425–432. 10.1016/j.dmpk.2016.08.007 27836712

[B27] Khokhar J. Y. Henricks A. M. Sullivan E. D. K. Green A. I. (2018). Unique effects of clozapine: a pharmacological perspective. Adv. Pharmacol. 82, 137–162. 10.1016/bs.apha.2017.09.009 29413518 PMC7197512

[B28] Liu T. Gao P. Xie C. Zhang H. Shi Z. Chen R. (2023). Study on the daily dose and serum concentration of clozapine in psychiatric patients and possible influencing factors of serum concentration. BMC Psychiatry 23, 596. 10.1186/s12888-023-05078-z 37582705 PMC10428656

[B29] Mauri M. C. Paletta S. Di Pace C. Reggiori A. Cirnigliaro G. Valli I. (2018). Clinical pharmacokinetics of atypical antipsychotics: an update. Clin. Pharmacokinet. 57, 1493–1528. 10.1007/s40262-018-0664-3 29915922

[B30] Mayerova M. Ustohal L. Jarkovsky J. Pivnicka J. Kasparek T. Ceskova E. (2018). Influence of dose, gender, and cigarette smoking on clozapine plasma concentrations. Neuropsychiatr. Dis. Treat. 14, 1535–1543. 10.2147/NDT.S163839 29950838 PMC6011879

[B31] Menkes D. B. Glue P. Gale C. Lam F. Hung C. T. Hung N. (2018). Steady-State clozapine and norclozapine pharmacokinetics in Maori and European patients. EBioMedicine 27, 134–137. 10.1016/j.ebiom.2017.11.030 29254680 PMC5828556

[B32] Molins C. Carceller-Sindreu M. Navarro H. Carmona C. Piñeiro M. Martínez E. (2017). Plasma ratio of clozapine to N-desmethylclozapine can predict cognitive performance in treatment-resistant psychotic patients. Psychiatry Res. 258, 153–157. 10.1016/j.psychres.2017.10.010 29024893

[B33] Ng C. H. Chong S.-A. Lambert T. Fan A. Peter Hackett L. Mahendran R. (2005). An inter-ethnic comparison study of clozapine dosage, clinical response and plasma levels. Int. Clin. Psychopharmacol. 20, 163–168. 10.1097/00004850-200505000-00007 15812267

[B34] Özdemir V. Kalow W. Posner P. Collins E. J. Kennedy J. L. Tang B.-K. (2001). CYP1A2 activity as measured by a caffeine test predicts clozapine and active metabolite steady-state concentrationin patients with schizophrenia. J. Clin. Psychopharmacol. 21, 398–407. 10.1097/00004714-200108000-00007 11476124

[B35] Patteet L. Maudens K. E. Vermeulen Z. Dockx G. De Doncker M. Morrens M. (2014). Retrospective evaluation of therapeutic drug monitoring of clozapine and norclozapine in Belgium using a multidrug UHPLC–MS/MS method. Clin. Biochem. 47, 336–339. 10.1016/j.clinbiochem.2014.09.021 25289972

[B36] Perrild H. Kayser L. Poulsen H. E. Skovsted L. Jørgensen B. Hansen J. M. (1989). Differential effect of continuous administration of beta-adrenoceptor antagonists on antipyrine and phenytoin clearance. Br. J. Clin. Pharmacol. 28, 551–554. 10.1111/j.1365-2125.1989.tb03541.x 2574053 PMC1380015

[B37] Piwowarska J. Radziwoń-Zaleska M. Dmochowska M. Szepietowska E. Matsumoto H. Sygitowicz G. (2016). The usefulness of monitored therapy using Clozapine concentration in the blood serum for determining drug dose in Polish schizophrenic patients. Pharmacol. Rep. 68, 1120–1125. 10.1016/j.pharep.2016.06.016 27588387

[B38] Polasek T. M. Lin F. P. Miners J. O. Doogue M. P. (2011). Perpetrators of pharmacokinetic drug-drug interactions arising from altered cytochrome P450 activity: a criteria-based assessment. Br. J. Clin. Pharmacol. 71, 727–736. 10.1111/j.1365-2125.2011.03903.x 21223357 PMC3093078

[B39] Pollock B. G. (1997). Gender differences in psychotropic drug metabolism. Psychopharmacol. Bull. 33, 235–241. 9230636

[B40] Raedler T. J. Hinkelmann K. Wiedemann K. (2008). Variability of the *in vivo* metabolism of clozapine. Clin. Neuropharmacol. 31, 347–352. 10.1097/WNF.0b013e31815cba61 19050412

[B41] Rajkumar A. P. Poonkuzhali B. Kuruvilla A. Jacob M. Jacob K. S. (2013). Clinical predictors of serum clozapine levels in patients with treatment-resistant schizophrenia. Int. Clin. Psychopharmacol. 28, 50–56. 10.1097/YIC.0b013e32835ac9da 23104241

[B42] Remington G. Lee J. Agid O. Takeuchi H. Foussias G. Hahn M. (2016). Clozapine’s critical role in treatment resistant schizophrenia: ensuring both safety and use. Expert Opin. Drug Saf. 15, 1193–1203. 10.1080/14740338.2016.1191468 27207070

[B43] Rostami-Hodjegan A. Amin A. M. Spencer E. P. Lennard M. S. Tucker G. T. Flanagan R. J. (2004). Influence of dose, cigarette smoking, age, sex, and metabolic activity on plasma clozapine concentrations: a predictive model and nomograms to aid clozapine dose adjustment and to assess compliance in individual patients. J. Clin. Psychopharmacol. 24, 70–78. 10.1097/01.jcp.0000106221.36344.4d 14709950

[B44] Russo M. Ignjatovic-Ristic D. Cohen D. Arenliu A. Bajraktarov S. Dzubur Kulenovic A. (2023). Clozapine prescription rates in Southeast Europe: a cross-sectional study. Front. Psychiatry 14, 1123246. 10.3389/fpsyt.2023.1123246 37113539 PMC10126685

[B45] Samara M. T. Dold M. Gianatsi M. Nikolakopoulou A. Helfer B. Salanti G. (2016). Efficacy, acceptability, and tolerability of antipsychotics in treatment-resistant schizophrenia: a network meta-analysis. JAMA Psychiatry 73, 199–210. 10.1001/jamapsychiatry.2015.2955 26842482

[B46] Scandlyn M. J. Stuart E. C. Rosengren R. J. (2008). Sex-specific differences in CYP450 isoforms in humans. Expert Opin. Drug Metabolism Toxicol. 4, 413–424. 10.1517/17425255.4.4.413 18524030

[B47] Schoretsanitis G. Kane J. M. Correll C. U. Marder S. R. Citrome L. Newcomer J. W. (2020). Blood Levels to Optimize Antipsychotic Treatment in Clinical Practice: a Joint Consensus Statement of the American Society of Clinical Psychopharmacology and the Therapeutic Drug Monitoring Task Force of the Arbeitsgemeinschaft für Neuropsychopharmakologie und Pharmakopsychiatrie. J. Clin. Psychiatry 81, 19cs13169. 10.4088/JCP.19cs13169 32433836

[B48] Schoretsanitis G. Smith R. L. Molden E. Solismaa A. Seppälä N. Kopeček M. (2021). European whites may need lower minimum therapeutic clozapine doses than those customarily proposed. J. Clin. Psychopharmacol. 41, 140–147. 10.1097/JCP.0000000000001341 33587398

[B49] Shin J. G. Soukhova N. Flockhart D. A. (1999). Effect of antipsychotic drugs on human liver cytochrome P-450 (CYP) isoforms *in vitro*: preferential inhibition of CYP2D6. Drug Metab. Dispos. 27, 1078–1084. 10460810

[B50] Silva Gracia M. Köppl A. Unholzer S. Haen E. (2017). Development and validation of an HPLC-UV method for the simultaneous determination of the antipsychotics clozapine, olanzapine and quetiapine, several beta-blockers and their metabolites. Biomed. Chromatogr. 31, e3968. 10.1002/bmc.3968 28266722

[B51] Siskind D. Mccartney L. Goldschlager R. Kisely S. (2016). Clozapine v. first- and second-generation antipsychotics in treatment-refractory schizophrenia: systematic review and meta-analysis. Br. J. Psychiatry 209, 385–392. 10.1192/bjp.bp.115.177261 27388573

[B52] Siwek M. (2015). Potential interaction-related toxicity of clozapine. Psychiatr. i Psychol. Klin. 15, 86–91. 10.15557/pipk.2015.0014

[B53] Siwek M. Woroń J. Gorostowicz A. Wordliczek J. (2020). Adverse effects of interactions between antipsychotics and medications used in the treatment of cardiovascular disorders. Pharmacol. Rep. 72, 350–359. 10.1007/s43440-020-00058-6 32124390

[B54] Smith S. (2010). Gender differences in antipsychotic prescribing. Int. Rev. Psychiatry 22, 472–484. 10.3109/09540261.2010.515965 21047160

[B55] Spina E. Hiemke C. De Leon J. (2016). Assessing drug-drug interactions through therapeutic drug monitoring when administering oral second-generation antipsychotics. Expert Opin. Drug Metabolism Toxicol. 12, 407–422. 10.1517/17425255.2016.1154043 26878495

[B56] Suhas S. Kumar V. Damodharan D. Sharma P. Rao N. P. Varambally S. (2020). Do Indian patients with schizophrenia need half the recommended clozapine dose to achieve therapeutic serum level? An exploratory study. Schizophrenia Res. 222, 195–201. 10.1016/j.schres.2020.05.057 32518001

[B57] Tang Y. L. Mao P. Li F. M. Li W. Chen Q. Jiang F. (2007). Gender, age, smoking behaviour and plasma clozapine concentrations in 193 Chinese inpatients with schizophrenia. Br. J. Clin. Pharmacol. 64, 49–56. 10.1111/j.1365-2125.2007.02852.x 17298477 PMC2000616

[B58] Wagner E. Mcmahon L. Falkai P. Hasan A. Siskind D. (2020). Impact of smoking behavior on clozapine blood levels - a systematic review and meta-analysis. Acta Psychiatr. Scand. 142, 456–466. 10.1111/acps.13228 32869278

[B59] Weiner D. M. Meltzer H. Y. Veinbergs I. Donohue E. M. Spalding T. A. Smith T. T. (2004). The role of M1 muscarinic receptor agonism of N-desmethylclozapine in the unique clinical effects of clozapine. Psychopharmacology 177, 207–216. 10.1007/s00213-004-1940-5 15258717

[B60] WHO (1993). The ICD-10 classification of mental and behavioural disorders. WHO. 10.1007/BF00788743 8284737

[B61] Wishart D. S. Feunang Y. D. Guo A. C. Lo E. J. Marcu A. Grant J. R. (2018). DrugBank 5.0: a major update to the DrugBank database for 2018. Nucleic Acids Res. 46, D1074–d1082. 10.1093/nar/gkx1037 29126136 PMC5753335

[B62] Zeng L. Lv H. Li J. Xue R. Liu X. Zhou C. (2022). Cigarette smoking, coffee consumption, alcohol intake, and clozapine metabolism: a Mendelian randomization study. Front. Psychiatry 13, 1002235. 10.3389/fpsyt.2022.1002235 36245885 PMC9559606

